# Epigenome-Wide Association Studies of the Fractional Exhaled Nitric Oxide and Bronchodilator Drug Response in Moderate-to-Severe Pediatric Asthma

**DOI:** 10.3390/biomedicines11030676

**Published:** 2023-02-23

**Authors:** Mario Martin-Almeida, Javier Perez-Garcia, Esther Herrera-Luis, Carlos Rosa-Baez, Mario Gorenjak, Anne H. Neerincx, Olaia Sardón-Prado, Antoaneta A. Toncheva, Susanne Harner, Christine Wolff, Susanne Brandstetter, Elisa Valletta, Mahmoud I. Abdel-Aziz, Simone Hashimoto, Vojko Berce, Paula Corcuera-Elosegui, Javier Korta-Murua, Heike Buntrock-Döpke, Susanne J. H. Vijverberg, Joris C. Verster, Nikki Kerssemakers, Anna M Hedman, Catarina Almqvist, Jesús Villar, Aletta D. Kraneveld, Uroš Potočnik, Michael Kabesch, Anke H. Maitland-van der Zee, Maria Pino-Yanes, on behalf of the SysPharmPediA Consortium

**Affiliations:** 1 Genomics and Health Group, Department of Biochemistry, Microbiology, Cell Biology, and Genetics, Universidad de La Laguna (ULL), 38200 San Cristóbal de La Laguna, Spain; 2 Center for Human Molecular Genetics and Pharmacogenomics, Faculty of Medicine, University of Maribor, 2000 Maribor, Slovenia; 3 Department of Respiratory Medicine, Amsterdam University Medical Centres—Loc. AMC, University of Amsterdam, 1105 AZ Amsterdam, The Netherlands; 4 Division of Pediatric Respiratory Medicine, Donostia University Hospital, 20014 San Sebastián, Spain; 5 Department of Pediatrics, University of the Basque Country (UPV/EHU), 48013 San Sebastián, Spain; 6 Department of Pediatric Pneumology and Allergy, University Children’s Hospital Regensburg (KUNO) at the Hospital St. Hedwig of the Order of St. John, University of Regensburg, D-93049 Regensburg, Germany; 7 Department of Clinical Pharmacy, Faculty of Pharmacy, Assiut University, Assiut 71515, Egypt; 8 Department of Pediatric Respiratory Medicine, Emma Children’s Hospital, Amsterdam UMC, 1105 AZ Amsterdam, The Netherlands; 9 Clinic of Pediatrics, University Medical Centre Maribor, 2000 Maribor, Slovenia; 10 Division of Pharmacology, Utrecht Institute for Pharmaceutical Sciences, Faculty of Science, Utrecht University, 3584 CG Utrecht, The Netherlands; 11 Centre for Human Psychopharmacology, Swinburne University, Melbourne, VIC 3122, Australia; 12 Department of Medical Epidemiology and Biostatistics, Karolinska Institutet, Solna, 171 77 Stockholm, Sweden; 13 CIBER de Enfermedades Respiratorias, Instituto de Salud Carlos III, 28029 Madrid, Spain; 14 Multidisciplinary Organ Dysfunction Evaluation Research Network, Research Unit, Hospital Universitario Dr. Negrín, 35010 Las Palmas de Gran Canaria, Spain; 15 Laboratory for Biochemistry, Molecular Biology, and Genomics, Faculty of Chemistry and Chemical Engineering, University of Maribor, 2000 Maribor, Slovenia; 16 Instituto de Tecnologías Biomédicas (ITB), Universidad de La Laguna (ULL), 38200 San Cristóbal de La Laguna, Spain

**Keywords:** epigenetic, biomarker, methylation, asthma, FeNO, BDR, precision medicine

## Abstract

Asthma is the most prevalent pediatric chronic disease. Bronchodilator drug response (BDR) and fractional exhaled nitric oxide (FeNO) are clinical biomarkers of asthma. Although DNA methylation (DNAm) contributes to asthma pathogenesis, the influence of DNAm on BDR and FeNO is scarcely investigated. This study aims to identify DNAm markers in whole blood associated either with BDR or FeNO in pediatric asthma. We analyzed 121 samples from children with moderate-to-severe asthma. The association of genome-wide DNAm with BDR and FeNO has been assessed using regression models, adjusting for age, sex, ancestry, and tissue heterogeneity. Cross-tissue validation was assessed in 50 nasal samples. Differentially methylated regions (DMRs) and enrichment in traits and biological pathways were assessed. A false discovery rate (FDR) < 0.1 and a genome-wide significance threshold of *p* < 9 × 10^−8^ were used to control for false-positive results. The CpG cg12835256 (*PLA2G12A*) was genome-wide associated with FeNO in blood samples (coefficient= −0.015, *p* = 2.53 × 10^−9^) and nominally associated in nasal samples (coefficient = −0.015, *p* = 0.045). Additionally, three CpGs were suggestively associated with BDR (FDR < 0.1). We identified 12 and four DMRs associated with FeNO and BDR (FDR < 0.05), respectively. An enrichment in allergic and inflammatory processes, smoking, and aging was observed. We reported novel associations of DNAm markers associated with BDR and FeNO enriched in asthma-related processes.

## 1. Introduction

Asthma is a heterogeneous respiratory disease that affects more than 330 million people worldwide and is the most prevalent chronic disease in children. Moreover, asthma is associated with one in every 250 deaths worldwide [1]. Characterized by chronic airway inflammation and variable expiratory airflow limitation, asthma is defined by its symptoms, including wheezing, shortness of breath, chest tightness, and cough, which may vary over time and in intensity. Anti-inflammatory and bronchodilator drugs are the main therapy to control asthma symptoms, improve lung function, and reduce the risk of severe exacerbations [2]. However, less than 5% of pediatric asthma patients have severe asthma, defined as uncontrolled asthma that often does not respond to the currently available medications despite adherence to maximal optimized high doses [3]. Severe asthma patients have the lowest quality of life and the highest risk for morbidity and mortality, and their treatment consumes the majority of healthcare resources due to asthma [3]. 

The fractional exhaled nitric oxide (FeNO) and the bronchodilator drug response (BDR) are two of the main clinical biomarkers that can be objectively measured for asthma diagnosis and for assessing treatment response. FeNO is the only available non-invasive biomarker for type 2 airway inflammation in asthma. It can be quantified in exhaled air of children and adults through portable and non-invasive devices. FeNO levels in exhaled air have been related to chronic inflammatory diseases, Th2-mediated immune responses, and airway eosinophilia [2]. BDR measures changes in lung function after administration of short-acting β_2_-agonists [4], the main rapid-onset airway bronchodilators. BDR is also associated with the severity and instability of asthma and is also frequently used for asthma phenotyping. FeNO and BDR are conditioned by genetic and environmental factors [5,6].

Precision medicine aims to improve the prevention and treatment of diseases based on features inherent to each patient [7]. The genetic background greatly contributes to asthma, estimating the heritability of asthma susceptibility over 55–90% and 70% to treatment response [7]. However, currently reported genetic variants only partially explain this heritability. Asthma is a multifactorial disease, and susceptibility and treatment response are influenced by genetic variation and environmental factors [7]. DNA methylation (DNAm), an epigenetic mechanism that reflects the interaction between both genetic and environmental factors, could explain part of the heritability of asthma treatment response [8]. Epigenome-wide association studies (EWAS) have identified promising CpG sites and differentially methylated regions (DMRs) involved in asthma [9,10]. Despite being useful clinical biomarkers in the management of asthma, the relationship of DNAm with FeNO and BDR has been scarcely investigated. Cardenas et al. [9] profiled DNAm in nasal cells in 547 children from different populations and identified a large set of CpGs and DMRs associated with multiple asthma-related phenotypes, including FeNO (8,372 CpGs and 191 DMRs with FDR < 0.05) and BDR (130 CpGs with an FDR < 0.05). They reported that FeNO-associated epigenetic markers were enriched in intracellular membrane trafficking, T cell activation, oxidative stress, mucin production, neutrophil degranulation, and interleukin pathways. Multiple DMRs were located in asthma-associated genes, including *TNIP-1*, *IL-13*, and *CHI3L1*. For the BDR trait, they did not report any significant DMRs and observed that the associated epigenetic markers did not overlap with any other asthma-related phenotype. However, this study only included 12% of individuals with current asthma. On the other hand, FeNO-related genes, including multiple nitric oxide synthase (*NOS*) and arginase (*ARG*) isoforms, have been evaluated in candidate-gene epigenetic studies, but controversial results of the effect of epigenetic markers in these genes and FeNO have been reported [11,12].

The association of FeNO and BDR with both environmental and genetic factors has been reported, but its relationship with the epigenome in asthma patients remains barely investigated. Thus, we hypothesize that DNAm from whole blood is a biomarker associated with BDR and FeNO that captures the influence of environmental and genetic factors on these clinical biomarkers. Therefore, this study aimed to identify DNAm markers in whole blood associated with BDR or FeNO in pediatric asthma, assess whether they are shared with nasal epithelium cells, and analyze their enrichment in previous associations and/or biological processes.

## 2. Materials and Methods

### 2.1. Study Population

This study was conducted in children with moderate-to-severe asthma from the Systems Pharmacology Approach to Uncontrolled Pediatric Asthma (SysPharmPediA) consortium (Clinicaltrials.gov ID: NCT04865575) [13]. This study was conducted following the Declaration of Helsinki and approved by the ethics committees of participating institutions: University Regensburg, Germany (18-1034-101); Clinical Research Ethics Committee of Basque Country, Spain [PI2015075 (SO)]; Medical Ethics Committee of the University Medical Center Utrecht (UMC Utrecht), Utrecht, The Netherlands (NL55788.041.15); National Medical Ethics Committee, Slovenia (0120-569/2017/4); and Ethical Regional Review Board in Stockholm, Sweden (Dnr 2010/1336-31/3 and 2019-00546). All parents and participants provided signed written consent and agreed to participate in the study. The study design and characteristics of the enrolled patients have already been described elsewhere [13]. Briefly, patients were enrolled in four European countries (Spain, The Netherlands, Slovenia, and Germany) according to the following inclusion criteria: (1) aged between 6 and 17 years old, (2) physician’s diagnosis of asthma, and (3) moderate-to-severe patients with asthma treated with medication step ≥ 3 according to the Global Initiative for Asthma (GINA) guidelines [2]. In the current study, only individuals with available data on BDR and/or FeNO were included.

Clinical and demographic variables of interest were recorded using standardized questionnaires. Asthma control was assessed based on (childhood) Asthma Control Test (ACT/cACT) and the development of asthma exacerbations. Uncontrolled asthma was defined as having an ACT/cACT score ≤ 19 or ≥ 1 exacerbation requiring oral corticosteroids or severe exacerbation requiring hospitalization or emergency room visits in the past year. Controlled asthma was defined as asthma patients with an ACT/cACT score > 19 and no severe exacerbations in the past year. Body mass index (BMI) was estimated as BMI = weight (kg)/height (m^2^). According to the World Health Organization (WHO) recommendations, we expressed BMI as z-scores [14].

### 2.2. Measurement of FeNO and BDR

Spirometry tests pre- and post-administration of bronchodilator and FeNO assessment were performed according to the European Respiratory Society/American Thoracic Society (ERS/ATS) guidelines [15,16], as previously described [13]. BDR was calculated based on the forced expiratory volume in the first second (FEV_1_), expressed in liters, as BDR = $\frac{\mathrm{postFEV}1-\mathrm{preFEV}1}{\mathrm{preFEV}1}$ × 100. FeNO values were reported as parts per billion (ppb).

### 2.3. Genome-Wide Methylation Assessment and Quality Control

Blood samples were collected in K-EDTA vacutainer tubes for subsequent downstream applications, including genotyping and methylation profiling. Genomic DNA was isolated using the FlexiGene DNA Kit (Qiagen) according to the manufacturer’s instructions. After concentration measurements and quality control (QC), the samples were equilibrated to 10 ng/µL, and a total of 500 ng DNA was utilized for bisulfite conversion. Next, methylation levels of 865,918 CpG sites across the whole genome were assessed using the Infinium Illumina MethylationEPIC BeadChip array (Illumina Inc., San Diego, CA, USA). Raw data QC was conducted using the ENmix package (1.28.8) [17] in R (4.1.2) [18]. First, we removed low-quality samples and probes. Methylation data points were defined as low-quality data if their signal did not differ from negative control (detection *p*-value > 1 × 10^−6^) and/or they were measured by < 3 beads. Based on that, low-quality CpG probes were defined as those having ≥ 5% of bad-quality data points across samples. Low-quality samples were defined as (1) the presence of ≥ 5% of bad quality data points across CpGs, (2) having total bisulfite intensity less than three standard deviations than bisulfite controls, and/or (3) outliers of bisulfite intensity or beta value distribution.

We used the out-of-band (oob) method for background noise correction. Bisulfite intensities were normalized by quantile normalization to reduce variability among arrays. The EPIC array uses two different dyes and types of probes; (i) Regression on Correlated Probes (RCP) and (ii) the Regression on Logarithm of Internal Control (RELIC) methods [19,20] to correct dye- and probe-type biases. Methylation intensities were used to estimate beta values. Furthermore, outlier methylation data points for each CpG, defined as values below/above three times the interquartile range (IQR) from the first/third quantiles, were set as missing values. CpGs and samples with a missing rate of ≥5% and ≥10%, respectively, were removed. The remaining missing values were imputed (k-nearest neighbor method). Moreover, we estimated the predicted sex based on methylation data and discarded one sample with sex discordance between the reported and predicted sex.

We removed first- and second-degree related individuals previously identified based on genotype data obtained with the Global Screening Array (GSA, Illumina Inc.). Since we included both males and females, we removed probes within sex chromosomes. Potential cross-sample contamination was assessed based on the distribution of beta-values of the control genotype probes included in the EPIC array. We used the ewastools R package [21] to identify potential cross-sample contaminated individuals and inspected the beta value distribution plots. Potential problematic probes that may capture other artifacts different than methylation were also filtered out. These include probes: (1) with a multimodal distribution of beta values, (2) cross-reactive or non-specific, which bind non-specifically to the target region of interest [22], and (3) potentially polymorphic probes defined as those containing a single nucleotide polymorphism (SNP) with a minor allele frequency (MAF) > 1% based on the Illumina v1.0 B4 manifest file at the CpG site or a single base extension. Finally, beta values were transformed into M-values for improved statistical properties [23].

### 2.4. Estimation of Cell-Type Heterogeneity and Potential Confounders

Cell-type heterogeneity is the major confounder in EWAS performed in primary tissues. In whole blood, DNAm captures the epigenetic states of a mixture of cell types. These distinct methylation profiles may lead to false discoveries when looking for their correlation with the phenotype of interest [24]. Therefore, cell-type heterogeneity in whole blood was captured using the Reference-Free Adjustment for Cell-Type composition (ReFACTor) [24] algorithm in GLINT 1.0.4, adjusting for sex, age, and the first six genotype principal components (PCs). This method estimates a set of PCs correlated with cell-type composition and could be included as covariates in subsequent analyses to correct this major confounder. A principal component regression analysis was used to test for association between multiple potential confounding variables and global DNAm.

### 2.5. Epigenome-Wide Association Study

We conducted an EWAS to identify single CpG sites across the genome associated with FeNO or BDR. The association between the methylation M-values and FeNO/BDR was tested through linear regression models using the limma v3.48.3 R package [25], adjusting for age, sex, ancestry (six genotype PCs), and the first six ReFACTor components. A false discovery rate (FDR) of 10% was used to correct for multiple comparisons. The *p*-value < 9 × 10^−8^ threshold was used to declare genome-wide significant association, as recommended for the EPIC array [26]. Genome inflation factor (λ) and quantile–quantile plots (Q-Q plots) of regression *p*-values were examined to inspect genomic inflation. Probes were annotated to the nearest gene based on the Illumina manifest file v1.0 B4 [27] and GREAT v4.0.4 [28]. Furthermore, we evaluated the robustness of the results by conducting sensitivity analyses adjusting for ICS dosage and recruiting center and stratified analysis by sex, age, and ethnicity. Potentially problematic probes were filtered out for subsequent analyses.

### 2.6. Cross-Tissue Validation

We attempted to evaluate the effect of the CpGs identified in whole blood samples in 62 nasal samples. Clinical data are summarized in Supplementary Table S1. Nasal epithelial cells were collected using sterile nasal swabs (Paul Böttger OHG, Bodenmais, Germany) and stored at −80 °C until processing. Genomic DNA was isolated using the MasterPure Complete DNA & RNA Purification Kit (Lucigen, Teddington, UK) according to the manufacturer’s instructions. Genome-wide DNAm profiling was carried out using the Illumina Infinium MethylationEPIC array. QC of DNAm data, cell-tissue heterogeneity assessment, and association analyses were conducted following the same procedures described for the blood samples. 

### 2.7. Differentially Methylated Regions

A DMR analysis was conducted to identify genomic regions of several CpG sites in which the global DNAm might be associated with our outcomes of interest. Two independent software, comb-p [29] and DMRcate [30], were used to identify DMRs associated with BDR and FeNO. Regions were identified based on CpGs with *p*-value < 0.05 (comb-p) or FDR ≤ 0.2 (DMRcate), and those CpGs located within 750 bp (comb-p) or 1000 bp (DMRcate) were combined into the same region. Only DMRs significant after correcting for multiple testing overlapping in both methods (adjusted *p*-value < 0.05) were retained. Comb-p was used to identify DMRs associated with BDR and FeNO in nasal samples using the same parameters as in the discovery phase.

### 2.8. Enrichment Analyses

Enrichment analysis was next performed to identify whether a set of CpGs or genes were more likely to be related to biological pathways, gene ontology terms, or different traits than by chance. The top 100 probes from the EWAS of FeNO and BDR were included in CpG-set enrichment analyses to identify an enrichment of CpGs previously associated with any traits through EWAS. These analyses were carried out using the EWAS Toolkit [31]. Furthermore, the genes to which these CpGs were annotated were included in gene-set enrichment analyses to identify enrichment in biological pathways and gene ontologies using Enrichr [32].

## 3. Results

### 3.1. Quality Control of DNAm Data and Assessment of Global DNAm Patterns

After QC, epigenomic data were available for 124 individuals and 773,260 CpGs from whole blood, and 55 individuals and 636,555 CpGs from nasal epithelia were available for subsequent analyses. The individuals and CpGs removed or flagged during the QC are summarized in Supplementary Table S2. The principal regression analysis plot showed that age, sex, ReFACTor principal components (capturing cell-heterogeneity), and ancestry had the most significant associations with global methylation patterns (Supplementary Figures S1 and S2). As a result, these variables were considered the most relevant confounders to be included as covariates in the association analyses. FeNO and BDR also showed significant associations with global methylation patterns. 

### 3.2. Study Population

A total of 109 and 121 asthma patients from SysPharmPediA with available FeNO and BDR and epigenomic data in blood were analyzed. Their main characteristics are summarized in Table 1. Briefly, for the 121 patients, the median age was 12.0 years old, and 62% were male. The median FeNO was 16.0 ppb (IQR = 9.0–38.0), and the median BDR was 4.2% (IQR = 0.6–11.3). Most children were European (79.3%), and 63.6% had uncontrolled asthma. Regarding lung function, the median predicted percentage values were 93.6% (IQR = 82.5–103.2) and 99.4% (IQR = 91.2–108.1) for pre-FEV_1_ and pre-forced vital capacity (pre-FVC), respectively. 

### 3.3. Epigenome-Wide Association Study

**Figure 1:**
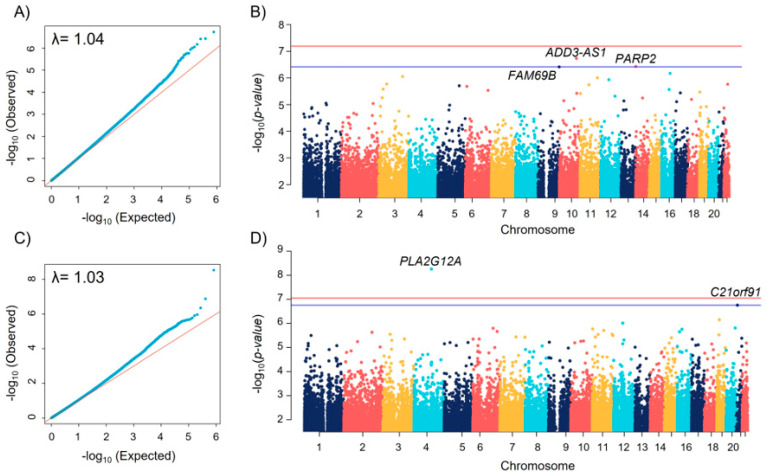
(**A**) Q-Q plot of *p*-values of the EWAS of BDR. (**B**) Manhattan plot for the EWAS of BDR. This figure illustrates the position of each CpG arranged by chromosomal location (X-axis) and the level of statistical significance measured by the negative logarithm of the corresponding *p*-value (Y-axis). The blue and red lines represent the FDR < 0.1 and genome-wide thresholds, respectively. (**C**) Q-Q plot of *p*-values of the EWAS of FeNO. (**D**) Manhattan plot for the EWAS of FeNO.

We did not observe genomic inflation both in the EWAS of BDR (λ = 1.04) (Figure 1A) and FeNO (λ = 1.03) (Figure 1C), indicating an optimal control for cell heterogeneity and other confounders. A total of three CpGs were significantly associated with BDR with FDR < 0.1 (Table 2, Figure 1B). The top hit was the CpG cg26203256, located in the *ADD3-AS1* gene, in which DNAm was associated with lower BDR (coefficient = −0.02, *p* = 1.85 × 10^−7^). On the other hand, the CpG cg12835256 located near *PLA2G12A* was genome-wide significantly associated with a reduction in FeNO (coefficient = −0.015, *p* = 2.53 × 10^−9^) (Table 2, Figure 1D). The negative coefficients for both associations suggest that these CpG are more likely to be hypomethylated in patients with higher FeNO or BDR values. These associations remained robust in all sensitivity analyses considering ICS dosage, recruiting centers, and ethnicity (Supplementary Table S3). The top-hit CpG cg12835256 (*PLA2G12A*) was cross-tissue associated in nasal samples (coefficient = −0.015, *p* = 0.045, Supplementary Table S4). This CpG is flagged in Illumina’s manifest file as a potential polymorphic probe. However, the potentially implicated SNP (rs4557260) is almost monomorphic in our population (minor allele count = 1).

Given that the age of the patients recruited in the study included puberty onset (13 years), where asthma prevalence trend switches between males and females [33,34], we also performed sensitivity analyses stratifying by sex and age. In the sex-stratified analysis, with the exception of the CpG from *C21orf91*, the rest of the CpGs were significant and showed consistent effects in both sexes (Supplementary Table S5). In the age-stratified analysis, all CpGs showed consistent effects in both age groups, but the one from *FAM69B* was not statistically significant in patients younger than 13 years old (Supplementary Table S5).

### 3.4. Differentially Methylated Regions

Four DMRs were associated with BDR using two independent software (adjusted *p*-value < 0.05) (Table 3). The top-hit was a region of 699 bp located in *C5orf63* (FDR = 2.80 × 10^−7^). The other DMRs were annotated to *PPFIBP2*, *RAMP1,* and *LY6G5C*. The top hit DMR located in *C5orf63* was cross-tissue validated in nasal samples (5: 126409007-5: 126409455, 9 CpGs, region *p*-value = 6.30 × 10^−7^, adjusted *p*-value = 9.54 × 10^−4^). In addition, 12 DMRs were associated with FeNO (adjusted *p*-value < 0.05) (Table 3). The top hit was a region of 1,458 bp located in *PPP5D1* (FDR = 1.48 × 10^−18^). The other DMRs were annotated to *EGR3*, *ODF3L1*, *HOXA6*, *HOXA-AS3*, *HOXA4*, *AURKC, MOGAT3*, *HOXA7*, *PDZRN4*, *MAGI2-AS3*, and *PTGS1.* The majority of DMRs showed a negative coefficient, indicating they are more likely hypomethylated in patients with higher FeNO or BDR values.

### 3.5. Enrichment Analyses

**Figure 2:**
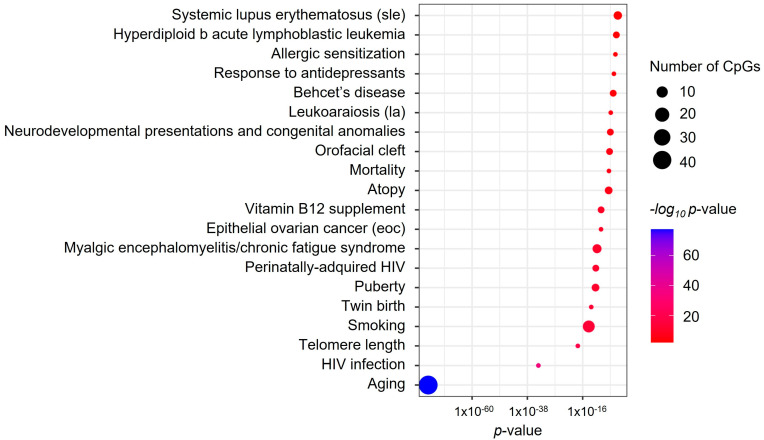
Summary plot of the trait-enrichment analysis for the top 100 CpGs associated with FeNO. *p*-values are displayed on the X-axis, and traits on the Y-axis. The color scale represents the significance of the association. The point size indicates the number of CpGs supporting the enrichment.

Regarding the EWAS of BDR, the gene-set enrichment analysis revealed enrichment in several pathways relevant to asthma, including IL-5 (FDR = 1.1 × 10^−2^), IL-2 (FDR = 4.2 × 10^−2^), Fc epsilon Receptor I signaling pathways (FDR = 3.3 × 10^−2^), and cellular aging (FDR = 4.2 × 10^−2^) (Supplementary Table S6). The CpG-set analyses showed enrichment in several traits, but they were supported only by one CpG (Supplementary Figure S3). The CpGs associated with FeNO demonstrated enrichment in previous EWAS associations, including aging (*p* = 1.33 × 10^−82^, 44 CpGs), smoking (*p* = 9.19 × 10^−16^, 13 CpGs), and puberty (*p* = 1.30 × 10^−11^, 6 CpGs) (Figure 2). The gene-set analysis only revealed the enrichment of genes implicated in the mechanism of action of the peroxisome proliferator-activated receptors (PPARs) (odds ratio [OR] = 138.18, FDR = 7.38 × 10^−3^, effect-causing genes: *PPARA* and *PTGS1*).

## 4. Discussion

This study investigated the role of DNAm in two clinical biomarkers, FeNO and BDR, in children with moderate-to-severe asthma. We identified one CpG located in the *PLA2G12A* gene as genome-wide significantly associated with FeNO and three CpGs significantly associated with BDR. Furthermore, twelve and four DMRs were related to FeNO and BDR, respectively. In addition, the top-hit CpG and DMR associated with FeNO and BDR, respectively, were cross-tissue validated in nasal samples. CpG-set and gene-set analyses revealed significant enrichment in previous EWAS signals and biological pathways involved in asthma. Our findings are relevant by providing new perspectives on the influence of DNAm on asthma biomarkers in pediatric asthma. Although some results overlap with a previous study (e.g., biological pathways such as interleukin signaling), our CpGs and differentially methylated sites and also its implications in biological pathways had not been previously reported by Cardenas et al. [9].

The CpG genome-wide, significantly associated with FeNO in whole blood and cross-validated in nasal samples (cg12835256), is located at the gene encoding for phospholipase A2 Group XIIA (*PLA2G12A*), a member of a group of phospholipases with a key role as regulators of type-2 inflammation, airway hyperresponsiveness, and eicosanoids production in asthma [35]. This enzyme, highly expressed in airway cells [36], acts as a high-affinity ligand for the PLA2R1 receptor, whose expression is elevated in children with asthma compared to healthy patients [37]. We also reported another CpG associated with FeNO, annotated to the chromosome 21 open reading frame 91 (*C21orf91*) gene. In a previous study, this gene was observed to be co-expressed with the IL-6 receptor (*IL-R6*), which has been associated with asthma risk. [38]. The complex IL-6/IL6R triggers a cascade that activates STAT3 and CEBP transcription factors, both involved in airway inflammation and asthma [39]. Increased levels of IL-6 have been related to impaired altered lung function and the use of high-dose ICS [40].

The most significant DMR for FeNO was annotated to *PPP5D1*, a pseudogene whose role in asthma is still unclear. Furthermore, we reported a DMR associated with FeNO located near *EGR3.* This gene encodes for a protein member of the early growth factors (EGR), which regulate the expression of genes related to inflammation and cell growth [41]. In addition, *EGR3* was reported to be overexpressed in the lung of asthma patients and regulates the expression of IL-2, IL-6, and IL-8 [42,43,44]. The other DMRs associated with FeNO are located in genes of the *HOXA* gene family (i.e., *HOXA-AS3*, *HOXA7*, and *HOXA4*), *AURKC*, *MAGI2-AS3*, *PDZRN4*, and *MOGAT3*. *HOXA-AS3* encodes a long noncoding RNA identified as a positive regulator of the necrosis factor kappa B (NF-κB) signaling [45]. NF-κB is a well-known transcription factor that leads to the expression of multiple pro-inflammatory mediators (e.g., cytokines and chemokines) involved in asthma [46]. In addition, *MOGAT3* encodes a protein that catalyzes the synthesis of diacylglycerol, a metabolite that attenuates the pathological consequences of inflammatory processes [47]. Furthermore, DNAm markers in *HOXA7* and *HOXA4* genes have been associated with lung development and asthma [48], while DNAm in *AURKC* has been associated with lung function in Latino children with asthma [49]. Finally, *MAGI2-AS3* encodes a lncRNA previously associated with asthma-related functions and pathways, such as cytokine production, cell proliferation, and kinase activity [50].

We reported the association of three epigenetic markers with BDR, located in *ADD3-AS1*, *PARP2*, and *FAM69B*. The *ADD3-AS1* gene is located in the antisense strand of *ADD3*, whose gene expression patterns correlate with serum immunoglobulin E (IgE) levels in atopic asthma patients [51]. The *PARP2* encodes a protein from PARP enzymes, which play key roles in asthma pathogenesis by affecting the expression of pro-inflammatory genes and chemokines [52]. Furthermore, we reported DMRs associated with BDR annotated to *C5orf63* (validated in nasal samples)*, PPFIBP2, LY6G5C,* and *RAMP1*. The receptor activity-modifying protein 1 (RAMP1) is a component of the Calcitonin gene-related protein (CGRP) receptor. Studies questioning the role of CGRP during allergic asthma have been contradictory, suggesting both protective (e.g., maintenance of vascular tone) and inflammation-promoting roles [53].

The epigenetic markers identified in this study showed enrichment in traits and biological processes, including PPARs, interleukins (IL-5 and IL-2), FcERI signaling pathways, smoking, and aging. PPARs have been established as critical components of type 2 immune response to allergens. The mechanism of action of PPARs is characterized by the ability to regulate lipid metabolism and inhibit proinflammatory transcription factors (e.g., NF-κB), which could make them a therapeutic target for asthma [54]. Patients with severe asthma have abnormal production of type 2 cytokines, whereas IL-5 is the main mediator of eosinophilic inflammation [55]. Similarly, alterations in the FcERI pathway are described in allergic diseases and associated with asthma exacerbations and allergic inflammation [56]. Interestingly, the strongest enrichment signal was related to aging. In the context of asthma, epigenetic age acceleration has been associated with asthma, allergic phenotypes, IgE, and FeNO [9,57]. In addition, we identified enrichment in CpGs related to smoking and second-hand smoke, which are well-known risk factors for asthma, impaired lung function, and unresponsiveness to corticosteroids [58].

We acknowledge some strengths of this work. Our study on pediatric patients provides a novel insight into a specific asthma phenotype that has been widely understudied: moderate-to-severe asthma. Moreover, we have controlled our analyses for known confounders in EWAS, such as age, sex, tissue heterogeneity, and ancestry. We also used microarrays that allow the agnostic genome-wide screening of DNAm changes with a low technical variation [59]. In addition, the multi-center pan-European nature makes the results likely generalizable to multiple populations. However, some limitations of this study must be acknowledged. First, the sample size of our study is modest in comparison to previous EWAS, not allowing us to detect DNAm changes with small effects. However, the prevalence of children with moderate to severe asthma is generally low, making this a unique cohort. Second, focusing on this specific phenotype of moderate-to-severe pediatric asthma could limit the generalizability of the results to other asthma phenotypes. Third, we have not included an independent population to replicate our findings, but we assessed cross-tissue validation in nasal samples. Fourth, although we assessed DNAm in whole blood and nasal samples, other asthma-relevant tissues remained unexplored. Fifth, although we adopted the standard approach of adjusting by cell heterogeneity, we acknowledge that this could have reduced the statistical power to detect DNAm changes related to eosinophilic or Th2 asthma. Sixth, although we addressed any potential bias in our results related to age and sex, we have not recorded sex hormone levels or the age of puberty onset to better classify the patients in sensitive and stratified analyses.

## 5. Conclusions

In conclusion, we reported novel associations of epigenetic markers in whole blood associated with FeNO and BDR in children with moderate-to-severe asthma. These markers were enriched in asthma-related pathways, such as aging, smoking, and inflammatory and allergic responses. Furthermore, these findings provide new insights into potential epigenetic biomarkers of asthma that could be used as predictors of prognosis or treatment response.

## Figures and Tables

**Table 1:** Clinical and demographic characteristics of the study population.

Characteristics	n	EWAS of BDR	n	EWAS of FeNO
Sex (male)	121	75 (62)	109	67 (61.5)
Age (years)	121	12.0 (9.8−14.0)	109	12.1 (9.8−14.0)
Ancestry	121		109	
African		4 (3.3)		2 (1.9)
Asian		2 (1.7)		1 (0.9)
European		96 (79.3)		88 (80.7)
Latin		9 (7.4)		8 (7.3)
Mixed/Other		10 (8.3)		10 (9.2)
Body mass index (z-score)	121	0.5 (−0.3−4.0)	108	0.4 (-0.4−1.3)
Uncontrolled asthma	121	77 (63.6)	109	71 (65.1)
pre-FEV_1_ (predicted %)	121	93.6 (82.5−103.2)	108	95.3 (82.7−103.2)
pre-FVC (predicted %)	121	99.4 (91.2−108.1)	108	100.3 (91.0−107.6)
BDR (%)	121	4.2 (0.6−11.3)	108	4.2 (0.6−10.5)
FeNO (ppb)	120	16.0 (9.0−38.0)	109	16.0 (9.0−41.0)
SABAs	103	94 (91.3)	92	84 (91.3)
LABAs	103	96 (93.2)	92	87 (94.6)
ICS	103	103 (100)	92	92 (100)
LTRA	103	17 (16.5)	92	15 (16.3)
OCS	103	2 (1.9)	92	1 (1.09)
Biological therapy †	103	9 (8.7)	92	8 (8.7)

Categorical variables are described as counts (percentage), and continuous variables as median (interquartile range). † Biological therapy: Mepolizumab or Omalizumab intake. Abbreviations: FEV1: Forced Expiratory Volume in the first second; FVC: forced vital capacity; BDR: Bronchodilator drug response; FeNO: Fractional exhaled nitric oxide; SABA: Short-Acting Beta-Agonists; LABAs: Long-Acting Beta-Agonists; ICS: Inhaled corticosteroids; LTRA: Leukotriene Receptor Antagonists; OCS: Oral corticosteroids.

**Table 2:** Summary results of the epigenome-wide association studies.

CpG	Chr	Position ^†^	Gene	Relative Position *	Coef	SE	*p*-Value	FDR
**Bronchodilator drug response**								
cg26203256	10	111756055	*ADD3-AS1*	First intron	−0.020	0.004	1.85 × 10^−7^	0.099
cg14985321	14	20823915	*PARP2*	Tenth exon	−0.010	0.002	3.71 × 10^−7^	0.099
cg06975120	9	139606856	*FAM69B*	165 bp upstream	−0.034	0.006	3.86 × 10^−7^	0.099
**Fractional exhaled nitric oxide**								
cg12835256	4	110651671	*PLA2G12A*	439 bp upstream	−0.015	0.002	2.53 × 10^−9^	0.002
cg19644580	21	19166676	*C21orf91*	Fourth intron	0.006	0.001	1.29 × 10^−7^	0.050

^†^ Position based on GRCh37/hg19 build. * Relative position to the transcription start sites of each gene. Abbreviations. Chr: chromosome; Coef: Coefficient expressed as log2(fold-change); SE: Standard error; FDR: False discovery rate.

**Table 3:** Differentially methylated regions associated with BDR and FeNO.

Chr	Start ^†^	End ^†^	Gene	Relative Position *	N° of CpGs	Coefficient	FDR
**Bronchodilator drug response**							
5	126408755	126409453	*C5orf63*	Promoter, first exon, and intron	11	−0.024	2.80 × 10^−7^
11	7597813	7598150	*PPFIBP2*	Gene body (alternative transcripts)	5	−0.011	9.24 × 10^−7^
2	238767350	238767779	*RAMP1*	671 bp upstream	4	−0.022	2.67 × 10^−6^
6	31650734	31650849	*LY6G5C*	Gene body (alternative transcripts)	3	−0.023	8.96 × 10^−6^
**Fractional exhaled nitric oxide**							
19	46998382	46999839	*PPP5D1*	Third intron	16	−0.004	1.48 × 10^−18^
8	22560921	22561950	*EGR3*	10,622 bp upstream	7	−0.005	3.15 × 10^−17^
15	76016128	76016334	*ODF3L1*	Promoter and first exon	6	−0.003	6.66 × 10^−14^
7	27186553	27187559	*HOXA6*	Promoter and gene body	11	0.005	2.01 × 10^−13^
7	27183273	27184736	*HOXA-AS3*	First intron	33	0.004	2.53 × 10^−13^
7	27170240	27170831	*HOXA4*	Promoter and first exon	10	−0.006	1.00 × 10^−10^
19	57742111	57742443	*AURKC*	Promoter and first exon	10	−0.008	3.95 × 10^−10^
7	100844059	100844444	*MOGAT3*	First exon	7	−0.002	4.21 × 10^−10^
7	27198188	27198428	*HOXA7*	1857 bp upstream	3	0.005	1.37 × 10^−9^
12	41581774	41582136	*PDZRN4*	Promoter and first exon	6	−0.004	1.37 × 10^−9^
7	79083996	79084165	*MAGI2-AS3*	Second intron	7	0.003	2.59 × 10^−9^
9	125137543	125137593	*PTGS1*	Second intron	3	0.003	7.61 × 10^−9^

^†^ Position based on GRCh37/hg19 build. * Relative position to the transcription start sites. Abbreviations. Chr: Chromosome; BDR: bronchodilator drug response; FeNO: fractional exhaled nitric oxide.

## Data Availability

All data supporting the findings and conclusions of this study are reported and available in the main text and/or supplementary material of this article. For clinical and other omics data generated within the SysPharmPediA study, the authors will make these data available upon specific requests subject to the requestor obtaining ethical, research, data access, and collaboration approvals from the SysPharmPediA study management board. Requests can be sent to mdelpino@ull.edu.es and a.h.maitland@amsterdamumc.nl.

## Supplementary Materials

The following supporting information can be downloaded at: https://www.mdpi.com/article/10.3390/biomedicines11030676/s1, Table S1: Clinical and demographic characteristics of the individuals with methylation measured in nasal samples; Table S2: Summary table of the quality control of methylation data; Table S3: Sensitivity analysis for the main EWAS findings; Table S4: Evaluation of the effect of CpGs in nasal epithelial cells; Table S5: Summary results of sensitivity analyses stratifying by sex and age; Table S6: Summary results of the gene-set enrichment analysis for the EWAS of BDR; Figure S1: Principal component regression analysis plot of blood samples; Figure S2: Principal component regression analysis plot of nasal samples; Figure S3: CpG-set enrichment analysis plot for the top 100 CpGs associated with BDR. Supplementary SI: SysPharmPediA consortium members (authors list); Supplementary SII: Acknowledgements.
