# Supplementary material for: Epigenome-Wide Association Studies of the Fractional Exhaled Nitric Oxide and Bronchodilator Drug Response in Moderate-to-Severe Pediatric Asthma
Source: Biomedicines. 2023 Feb 23;11(3):676. doi: 10.3390/biomedicines11030676 (PMC10044864; doi:10.3390/biomedicines11030676)
Supplement: Supplementary file 1 [file biomedicines-11-00676-s001.zip › Supplementary Information.pdf]

Supplementary Information

Epigenome-Wide Association Studies of the Fractional Exhaled Nitric Oxide and Bronchodilator Drug Response in Moderate-to-Severe Pediatric Asthma

Mario Martin-Almeida [1,†], Javier Perez-Garcia [1,†], Esther Herrera-Luis [1], Carlos Rosa-Baez [1], Mario Gorenjak [2], Anne H. Neerincx [3], Olaia Sardón-Prado [4,5], Antoaneta A. Toncheva [6], Susanne Harner [6], Christine Wolff [6], Susanne Brandstetter [6], Elisa Valletta [6], Mahmoud I. Abdel-Aziz [3,7], Simone Hashimoto [3,8], Vojko Berce [2,9], Paula Corcuera-Elosegui [4], Javier Korta-Murua [4,5], Heike Buntrock-Döpke [6], Susanne J. H. Vijverberg [3], Joris C. Verster [10,11], Nikki Kerssemakers [10], Anna M Hedman [12], Catarina Almqvist [12], Jesús Villar [13,14], Aletta D. Kraneveld [10], Uroš Potočnik [2,9,15], Michael Kabesch [6], Anke H. Maitland-van der Zee [3,8], Maria Pino-Yanes [1,13,16,*] and on behalf of the SysPharmPediA Consortium [‡]

[1] Genomics and Health Group, Department of Biochemistry, Microbiology, Cell Biology, and Genetics, Universidad de La Laguna (ULL), 38200 San Cristóbal de La Laguna, Spain; alu0101103389@ull.edu.es (M.M.-A.); jpegarci@ull.edu.es (J.P.-G.); estherhrl@outlook.com (E.H.-L.); alu0101202372@ull.edu.es (C.R.-B.)

[2] Center for Human Molecular Genetics and Pharmacogenomics, Faculty of Medicine, University of Maribor, 2000 Maribor, Slovenia; mario.gorenjak@um.si (M.G.); vojko.berce@guest.arnes.si (V.B.); uros.potocnik@um.si (U.P.)

[3] Department of Respiratory Medicine, Amsterdam University Medical Centres—Loc. AMC, University of Amsterdam, 1105 AZ Amsterdam, The Netherlands; a.vanstuyvenbergneerincx@amsterdamumc.nl (A.H.N.); m.i.ibrahim@amsterdamumc.nl (M.I.A.-A.); s.hashimoto@amsterdamumc.nl (S.H.); s.j.vijverberg@amsterdamumc.nl (S.J.H.V.); a.h.maitland@amsterdamumc.nl (A.H.M.-v.d.Z.)

[4] Division of Pediatric Respiratory Medicine, Donostia University Hospital, 20014 San Sebastián, Spain; olaia.sardonprado@osakidetza.eus (O.S.-P.); paula.corcueraelosegui@osakidetza.eus (P.C.-E.); josejavier.kortamurua@osakidetza.eus (J.K.-M.)

[5] Department of Pediatrics, University of the Basque Country (UPV/EHU), 48013 San Sebastián, Spain

[6] Department of Pediatric Pneumology and Allergy, University Children's Hospital Regensburg (KUNO) at the Hospital St. Hedwig of the Order of St. John, University of Regensburg, D-93049 Regensburg, Germany; antoanetatoncheva@gmail.com (A.A.T.); susanne.harner@barmherzige-regensburg.de (S.H.); christine.wolff@barmherzige-regensburg.de (C.W.); susanne.brandstetter@barmherzige-regensburg.de (S.B.); elisa.valletta@barmherzige-regensburg.de (E.V.); heike.buntrock-doepke@barmherzige-regensburg.de (H.B.-D.); michael.kabesch@barmherzige-regensburg.de (M.K.)

[7] Department of Clinical Pharmacy, Faculty of Pharmacy, Assiut University, Assiut 71515, Egypt

[8] Department of Pediatric Respiratory Medicine, Emma Children's Hospital, Amsterdam UMC, 1105 AZ Amsterdam, The Netherlands

[9] Clinic of Pediatrics, University Medical Centre Maribor, 2000 Maribor, Slovenia

[10] Division of Pharmacology, Utrecht Institute for Pharmaceutical Sciences, Faculty of Science, Utrecht University, 3584 CG Utrecht, The Netherlands; j.c.verster@uu.nl (J.C.V.); n.kerssemakers@students.uu.nl (N.K.); a.d.kraneveld@uu.nl (A.D.K.)

[11] Centre for Human Psychopharmacology, Swinburne University, Melbourne, VIC 3122, Australia

[12] Department of Medical Epidemiology and Biostatistics, Karolinska Institutet, Solna, 171 77 Stockholm, Sweden; anna.hedman@ki.se (A.M.H.); catarina.almqvist@ki.se (C.A.)

[13] CIBER de Enfermedades Respiratorias, Instituto de Salud Carlos III, 28029 Madrid, Spain; jesus.villar54@gmail.com (J.V.)
[14] Multidisciplinary Organ Dysfunction Evaluation Research Network, Research Unit, Hospital Universitario Dr. Negrín, 35010 Las Palmas de Gran Canaria, Spain
[15] Laboratory for Biochemistry, Molecular Biology, and Genomics, Faculty of Chemistry and Chemical Engineering, University of Maribor, 2000 Maribor, Slovenia
[16] Instituto de Tecnologías Biomédicas (ITB), Universidad de La Laguna (ULL), 38200 San Cristóbal de La Laguna, Spain
* Correspondence: mdelpino@ull.edu.es; Tel: +34-9223-16502-6343
† These authors contributed equally to this work and share the first authorship.
‡ The SysPharmPediA consortium authors are listed in the Supplementary SI of this manuscript.

This Supplementary Information file contains the following items:

Table of contents	Page
**Supplementary Tables**	
Table S1. Clinical and demographic characteristics of the individuals with methylation measured in nasal samples.	3
Table S2. Summary table of the quality control of methylation data.	4
Table S3. Sensitivity analysis for the main EWAS findings.	5
Table S4. Evaluation of the effect of CpGs in nasal epithelial cells.	6
Table S5. Summary results of sensitivity analyses stratifying by sex and age.	7
Table S6. Summary results of the gene-set enrichment analysis for the EWAS of BDR.	8
**Supplementary Figures**	
Figure S1. Principal component regression analysis plot of blood samples.	9
Figure S2. Principal component regression analysis plot of nasal samples.	10
Figure S3. CpG-set enrichment analysis plot for the top 100 CpGs associated with BDR.	11

SUPPLEMENTARY TABLES

Supplementary Table S1. Clinical and demographic characteristics of the individuals with nasal samples.

Characteristics	n	BDR	n	FeNO
Sex (male)	52	34 (65.4)	48	32 (66.7)
Age (years)	52	12.1 (10.4–14)	48	12 (10.4–14)
Ancestry	51		47	
African		2 (3.9)		2 (4.3)
Asian		1 (2)		1 (2.1)
European		43 (84.3)		39 (83)
Latin		3 (5.9)		3 (6.3)
Mixed/other		2 (3.9)		2 (4.3)
Body mass index (z-score)	52	0.6 (-0.3–1.3)	48	0.6 (-0.4–1)
Uncontrolled asthma	52	44 (84.6)	48	41 (85.4)
pre-FEV_1 (predicted %)	52	91.7 (81–101.7)	48	91.6 (80.9–103.2)
pre-FVC (predicted %)	52	99.8 (91.7–107.8)	48	100.3 (91.6–108.9)
BDR (%)	52	6 (0.6–13)	48	5.2 (0.5-12.3)
FeNO (ppb)	51	13.3 (10.4–45.7)	48	14.8 (10.4–45.7)
SABAs	46	40 (87)	42	36 (90)
LABAs	46	44 (95.7)	42	40 (95.2)
ICS	46	46 (100)	42	42 (100)
LTRA	46	12 (26.1)	42	12 (28.6)
OCS	46	1 (2.2)	42	1 (2.4)
Biological therapy[†]	46	5 (10.9)	42	5 (11.9)

Categorical variables are described as counts (percentage) and continuous variables as median (interquartile range). [†] Biological therapy: Mepolizumab or Omalizumab intake**. Abbreviations:** FEV_1: Forced Expiratory Volume in the first second; FVC: forced vital capacity; BDR: Bronchodilator drug response; FeNO: Fractional exhaled nitric oxide; SABA: Short-Acting Beta-Agonists; LABAs: Long-Acting Beta-Agonists; ICS: Inhaled corticosteroids; LTRA: Leukotriene Receptor Antagonists; OCS: Oral corticosteroids.

Supplementary Table S2. Summary of the quality control.

Tissue	Blood	Nasal
**CpG sites**		
Initial number	865,918	866,297
Low quality probes	21,683	46,843
Probes within the sexual chromosomes (X and Y)	18,648	17,893
Multimodal probes	12,168	85,589
Cross-reactive/non-specific probes	40,870	43,254
SNP at the CpG site	24,007	24,208
SNP at single base extension from a CpG site	11,552	11,900
Probes capturing SNPs	55	55
Remaining number of CpGs available for analysis	773,260	636,555
**Quality control of individuals**		
Initial number	130	62
Outlier or bad quality samples	1	3
Individuals with sex discordance	1	1
Individual with mixed genotype distributions on the control SNP probes	0	2
Related individuals	4	1
Total remaining samples	124	55
Individuals with BDR and methylation data	121	55
Individuals with FeNO and methylation data	109	50

Abbreviations: SNP: Single Nucleotide Polymorphism; BDR: Bronchodilator Drug Response; FeNO: Fractional exhaled Nitric Oxide.

Supplementary Table S3. Sensitivity analysis for the main EWAS findings.

CpG	Chr	Position†	Gene	Main Analysis* n	Coef	SE	P	Adjusted by ICS dosage* n	Coef	SE	P	Adjusted by recruiting center* n	Coef	SE	P	Only Europeans* n	Coef	SE	P
**FeNO**																			
cg12835256	4	110651671	*PLA2G12A*	109	0.002	$2.53x10^{-9}$	0.002	92	-0.015	0.002	$2.22x10^{-8}$	109	-0.015	0.002	$1.32x10^{-8}$	88	-0.019	0.003	$6.73x10^{-10}$
cg19644580	21	19166676	*C21orf91*		0.001	$1.29x10^{-7}$	0.05		0.006	0.001	$7.93x10^{-7}$		0.006	0.001	$3.72x10^{-7}$		0.003	0.001	$6.96x10^{-3}$
**BDR**																			
cg06975120	9	139606856	*FAM69B*		0.006	$3.86x10^{-7}$	0.01		-0.037	0.007	$6.13x10^{-7}$		-0.019	0.004	$1.89x10^{-6}$		-0.037	0.007	$2.02x10^{-7}$
cg14985321	14	20823915	*PARP2*	122	0.002	$3.71x10^{-7}$	0.01	103	-0.010	0.002	$1.28x10^{-5}$	122	-0.010	0.002	$6.02x10^{-6}$	97	-0.011	0.002	$1.50x10^{-6}$
cg26203256	10	111756055	*ADD3-AS1*		0.004	$1.85x10^{-7}$	0.01		-0.021	0.004	$3.28x10^{-7}$		-0.037	0.006	$9.06x10^{-8}$		-0.020	0.004	$3.15x10^{-6}$

†Position based on GRCh37/hg19 build. *All models were also adjusted by age, sex, ethnicity, and tissue heterogeneity. **Abbreviations.** Coef: Coefficient expressed as log2(fold-change); SE: Standard error.

Supplementary Table S4. Evaluation of the effect of CpGs in nasal epithelial cells.

CpG	Chromosome	Position†	Gene	Coef	SE	*p*-value
**Bronchodilator drug response**						
cg26203256	10	111756055	*ADD3-AS1*	NA	NA	NA
cg14985321	14	20823915	*PARP2*	0.0001	0.004	0.984
cg06975120	9	139606856	*FAM69B*	0.005	0.009	0.564
**Fractional exhaled nitric oxide**						
cg12835256	4	110651671	*PLA2G12A*	-0.015	0.007	0.045
cg19644580	21	19166676	*C21orf91*	0.006	-0.007	0.406

†Position based on GRCh37/hg19 build. **Abbreviations**. Coef: Coefficient expressed as log2(fold-change); SE: Standard error. NA: not available.

0

Supplementary Table S5. Summary results of sensitivity analyses stratifying by sex and age.

				All individuals					<13 years old				≥13 years old				Males only				Females only		
**CpG**	**Chr**	**Pos**	**Gene**	**n**	**logFC**	**SE**	**P**	**FDR**	**n**	**logFC**	**SE**	**P**	**n**	**logFC**	**SE**	**P**	**n**	**logFC**	**SE**	**P**	**n**	**logFC**	**SE**	**P**
**FeNO**																								
cg12835256	4	110651671	*PLA2G12A*	109	-0.015	0.002	2.53×10^{-9}	0.002	64	-0.016	0.003	7.30×10^{-6}	45	-0.013	0.004	0.003	67	-0.014	0.004	0.001	42	-0.016	0.003	1.86x10-5
cg19644580	21	19166676	*C21orf91*		0.006	0.001	1.29×10^{-7}	0.050		0.006	0.002	4.09×10^{-4}		0.004	0.001	0.016		0.001	0.001	0.363		0.008	0.002	8.37x10-5
**BDR**																								
cg06975120	9	139606856	*FAM69B*	122	-0.034	0.006	3.86×10^{-7}	0.099	71	-0.015	0.009	0.116	51	-0.053	0.009	9.13×10^{-7}	76	-0.017	0.007	0.018	46	-0.082	0.012	2.66×10^{-8}
cg14985321	14	20823915	*PARP2*		-0.010	0.002	3.71×10^{-7}	0.099		-0.010	0.003	0.002		-0.014	0.003	1.18×10^{-4}		-0.011	0.003	1.07×10^{-4}		-0.012	0.004	0.009
cg26203256	10	111756055	*ADD3-AS1*		-0.020	0.004	1.86×10^{-7}	0.099		-0.024	0.005	3.14×10^{-5}		-0.017	0.006	0.008		-0.021	0.005	3.23×10^{-4}		-0.022	0.006	4.92×10^{-4}

Abbreviations: logFC: log2(fold-change); SE: standard error; FDR: false discovery rate.

Supplementary Table S6. Summary results of the gene-set enrichment analysis for the EWAS of BDR.

Term	*q*-value	Odds Ratio	Genes
**Pathways**			
**WikiPathway 2021 Human**			
IL-5 signaling pathway WP127	0.011	23	*LYN, SYK, KRAS, SOS1*
**KEGG 2021 Human**			
C-type lectin receptor signaling pathway	0.031	10.5	*SYK, PRKCD, BCL3, CBLB, KRAS*
Fc epsilon RI signaling pathway	0.033	12.9	*LYN, SYK, KRAS, SOS1*
B cell receptor signaling pathway	0.043	10.7	*LYN, SYK, KRAS, SOS1*
**Biocarta 2016**			
Fc Epsilon Receptor I Signaling in Mast Cells Homo sapiens	0.017	23.6	*LYN, SYK, SOS1*
BCR Signaling Pathway Homo sapiens	0.017	21.9	*LYN, SYK, SOS1*
IL 2 signaling pathway Homo sapiens	0.042	33.8	*SYK, SOS1*
Telomeres, Telomerase, Cellular Aging, and Immortality Homo sapiens	0.042	31.2	*XRCC6, KRAS*
**Ontologies**			
**GO Biological Process 2021**			
Positive regulation of protein dephosphorylation (GO:0035307)	0.031	27.6	*MAGI2, PRKCD, CAMTA1, DUSP26*

Abbreviations: GO: Gene Ontology.

SUPPLEMENTARY FIGURES

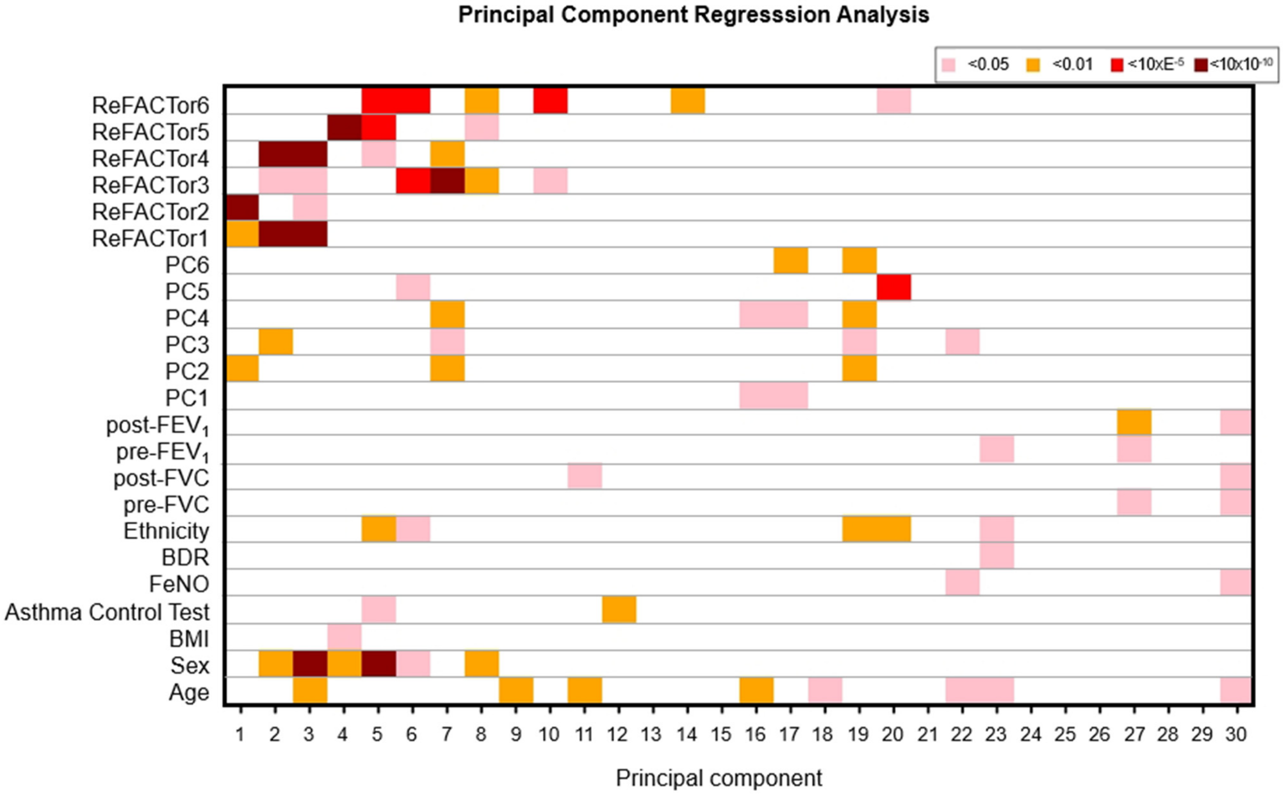

Supplementary Figure S1. Principal component regression analysis plot of blood samples. Top principal components are displayed along the X-axis, and covariates are displayed on the Y-axis. It shows with colors different levels of association p-values between principal components (PCs) and covariates. **Abbreviations:** PC: Principal component of genotype data; FEV_1: Forced expiratory volume in the first second; FVC: Forced vital capacity; FeNO: Fractional exhaled nitric oxide; BDR: Bronchodilator drug response; BMI: Body mass index.

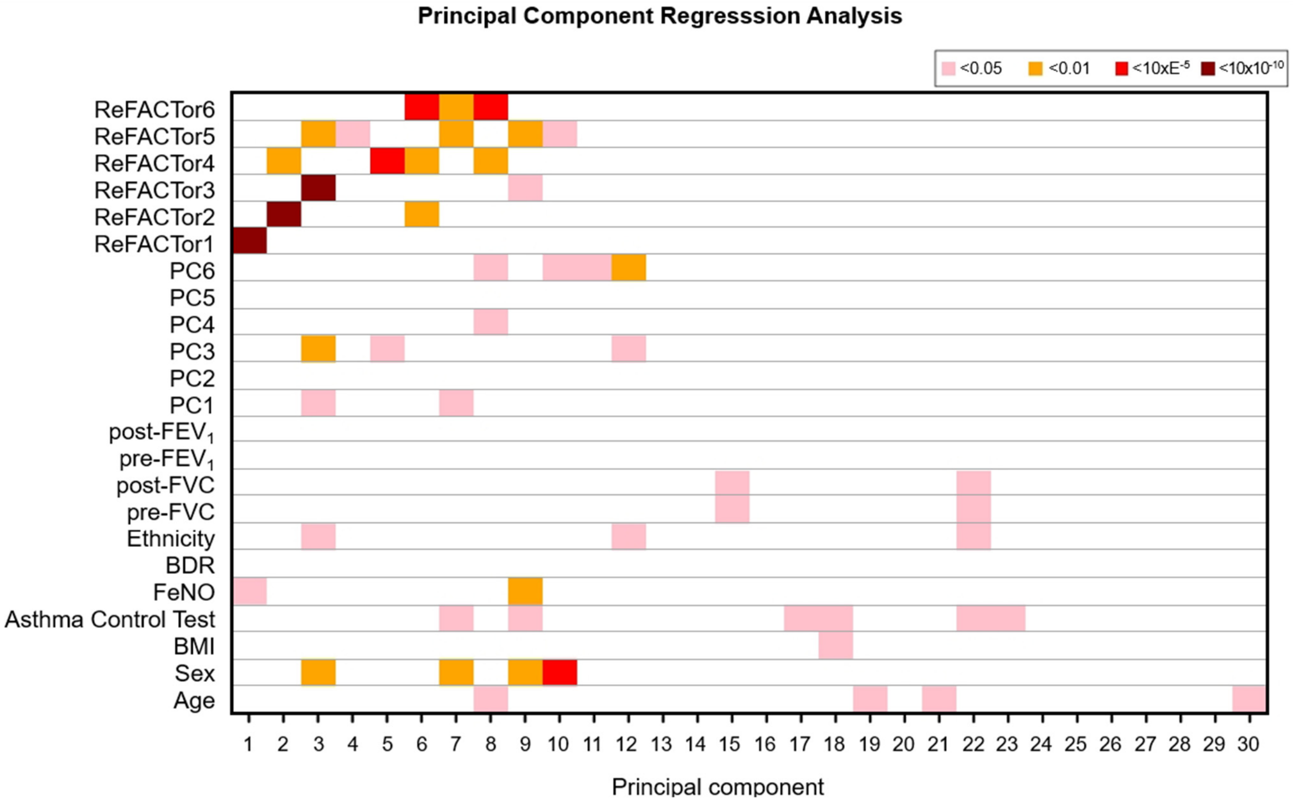

Supplementary Figure S2. Principal component regression analysis plot of nasal samples. Top principal components are displayed along the X-axis, and covariates are displayed on the Y-axis. It shows with colors different levels of association p-values between principal components (PCs) and covariates. **Abbreviations:** PC: Principal component of genotype data; FEV_1: Forced expiratory volume in the first second; FVC: Forced vital capacity; FeNO: Fractional exhaled nitric oxide; BDR: Bronchodilator drug response; BMI: Body mass index.

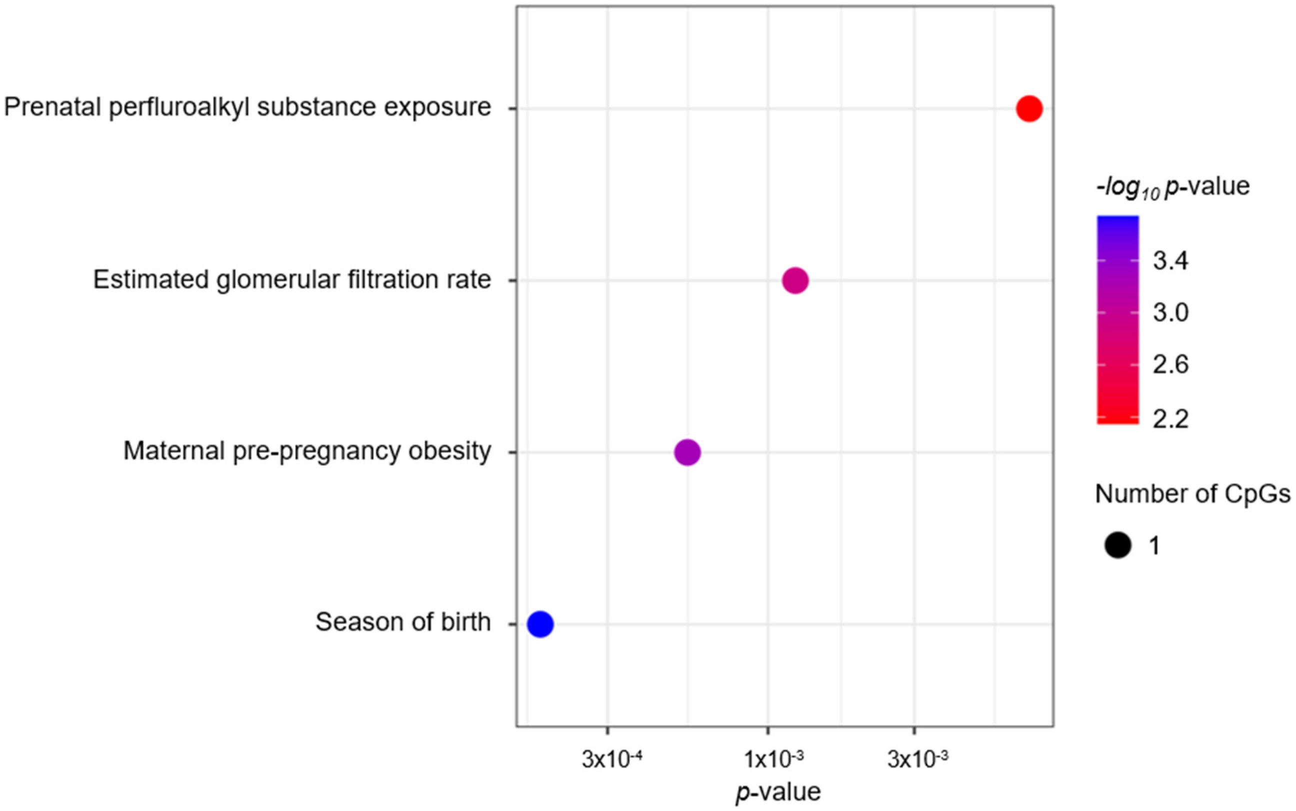

Supplementary Figure S3. Plot summarizing the trait-enrichment analysis for the top 100 CpGs for BDR in EWAS Atlas. *p*-values are displayed on the x-axis and traits on the y-axis. Blue shows the strongest associations, and the size of each point indicates the number of CpGs it correlates with.
