# Supplementary material for: Epigenome-Wide Association Studies of the Fractional Exhaled Nitric Oxide and Bronchodilator Drug Response in Moderate-to-Severe Pediatric Asthma
Source: Biomedicines. 2023 Feb 23;11(3):676. doi: 10.3390/biomedicines11030676 (PMC10044864; doi:10.3390/biomedicines11030676)
Supplement: Supplementary file 1 [file biomedicines-11-00676-s001.zip › Supplementary SII_SysPharmPediA_Acknowledgements_Feb_2023.pdf]

Supplementary SII: Acknowledgements

The SysPharmPediA consortium wishes to acknowledge the help and expertise of the following individuals and groups without whom, the study would not have been possible.

Others that contributed to SysPharmPediA:

Name	Country	Affiliation
Amaia Lorea Alvarez RN	Spain	Division of Pediatric Respiratory Medicine, Hospital Universitario Donostia, San Sebastián, Spain.
Arantzazu Zugasti Pérez RN	Spain	Division of Pediatric Respiratory Medicine, Hospital Universitario Donostia, San Sebastián, Spain.
Miren Lorea Otero Aramburu, NA	Spain	Division of Pediatric Respiratory Medicine, Hospital Universitario Donostia, San Sebastián, Spain.
María Isabel Gomez Osua RN[3]	Spain	Basque Biobank (Basque Biobank), Biodonostia Health Research Institute Association Node www.biobancovasco.org
Yennece W.F. Dagelet	The Netherlands	Department of Respiratory Medicine, Amsterdam UMC, University of Amsterdam, Amsterdam, The Netherlands.
Suzanne Terheggen-Lagro MD, PhD	The Netherlands	Department of Pediatric Respiratory Medicine, Emma Children's Hospital, Amsterdam UMC, Amsterdam, The Netherlands.
Niels W.P. Rutjes MD	The Netherlands	Department of Pediatric Respiratory Medicine, Emma Children's Hospital, Amsterdam UMC, Amsterdam, The Netherlands.
E. G. Haarman	The Netherlands	Department of Pediatric Respiratory Medicine, Emma Children's Hospital, Amsterdam UMC, Amsterdam, The Netherlands.
T. Dekker	The Netherlands	Department of Experimental Immunology, Academic Medical Center (AMC), University of Amsterdam, Amsterdam, The Netherlands.
B.S Dierdorp	The Netherlands	Department of Experimental Immunology, Academic Medical Center (AMC), University of Amsterdam, Amsterdam, The Netherlands.
R. Khurshid	The Netherlands	Department of Experimental Immunology, Academic Medical Center (AMC), University of Amsterdam, Amsterdam, The Netherlands.
B. Kapitein, MD, PhD	The Netherlands	Paediatric Intensive Care, Emma Children's Hospital, Amsterdam UMC, Amsterdam, The Netherlands.
G. Biesbroek	The Netherlands	Department of Pediatric Respiratory Medicine, Emma Children's Hospital, Amsterdam UMC, Amsterdam, The Netherlands.
J. Israels	The Netherlands	Department of Pediatric Respiratory Medicine, Emma Children's Hospital, Amsterdam UMC, Amsterdam, The Netherlands.
C. Brackel	The Netherlands	Department of Pediatric Respiratory Medicine, Emma Children's Hospital, Amsterdam UMC, Amsterdam, The Netherlands.
M. Verwaal – Maasstad	The Netherlands	Department of Pediatrics, Maasstad Hospital, Rotterdam, The Netherlands.
D. Seljogi	The Netherlands	Department of Pediatric Respiratory Medicine, Emma Children's Hospital, Amsterdam UMC, Amsterdam, The Netherlands.
P.F.M. Mau Asam	The Netherlands	Department of Respiratory Medicine, Amsterdam UMC, University of Amsterdam, Amsterdam, The Netherlands.
S. J.A. Lone-Latif	The Netherlands	Department of Respiratory Medicine, Amsterdam UMC, University of Amsterdam, Amsterdam, The Netherlands.
Davey Stigters	The Netherlands	Utrecht University, Master Pharmacy, Utrecht, The Netherlands.
Emine Sariguney	The Netherlands	Department of Respiratory Medicine, Amsterdam UMC, University of Amsterdam, Amsterdam, The Netherlands.
André Franke	Germany	Kiel

Juha Kere	Sweden/Finland	Stockholm/Helsinki
Anne Örtqvist	Sweden	Dept of Medical Epidemiology and Biostatistics, Karolinska Institutet, Stockholm, Sweden.
Vilhelmina Ullemar	Sweden	Dept of Medical Epidemiology and Biostatistics, Karolinska Institutet, Stockholm, Sweden.
Karin Dellenvall	Sweden	Dept of Medical Epidemiology and Biostatistics, Karolinska Institutet, Stockholm, Sweden.
Amir Hossein Alizadeh Bahmani, PharmD	The Netherlands	Amsterdam UMC location University of Amsterdam, Department of Pulmonary Medicine, Meibergdreef 9, Amsterdam, the Netherlands
Jelle M. Blankestijn, MSc	The Netherlands	Amsterdam UMC location University of Amsterdam, Department of Pulmonary Medicine, Meibergdreef 9, Amsterdam, the Netherlands
Shahriyar Shahbazi Khamas, PharmD	The Netherlands	Amsterdam UMC location University of Amsterdam, Department of Pulmonary Medicine, Meibergdreef 9, Amsterdam, the Netherlands
Natalia Hernandez-Pacheco, PhD	Sweden	Department of Clinical Science and Education, Södersjukhuset, Karolinska Institutet, Stockholm, Sweden. CIBER de Enfermedades Respiratorias, Instituto de Salud Carlos III, Madrid, Spain.
Fabian Lorenzo-Diaz, PhD	Spain	Genomics and Health Group, Department of Biochemistry, Microbiology, Cell Biology and Genetics, Universidad de La Laguna, La Laguna, Santa Cruz de Tenerife, Spain. Instituto de Enfermedades Tropicales y Salud Pública de Canarias (IETSPC), La Laguna, Tenerife, Spain.

Funding

The SysPharmPediA consortium is supported by ZonMW [project number: 9003035001], the Ministry of Education, Science, and Sport of the Republic of Slovenia [contract number C330-16-500106]; the German Ministry of Education and Research (BMBF) [project number FKZ 031L0088]; Instituto de Salud Carlos III (ISCIII) through Strategic Action for Health Research (AES) and European Community (EC) within the Active and Assisted Living (AAL) Program framework [award numbers AC15/00015 and AC15/00058] under the frame of the ERACoSysMed JTC-1 Call. MP-Y was also supported by a grant from the Ramón y Cajal Program (RYC-2015-17205) by MCIN/AEI/10.13039/501100011033 and by the European Social Fund “ESF Investing in your future”. JP-G was funded by the fellowship FPU19/02175 (Formación Profesorado Universitario Program) from the Spanish Ministry of Universities. EH-L was funded by fellowship PRE2018-083837 from MCIN/AEI /10.13039/501100011033 and the European Social Fund “Investing in your future”. MP-Y and JV were also supported by CIBER - Consorcio Centro de Investigación Biomédica en Red- (CIBERES), Instituto de Salud Carlos III (ISCIII), and European Regional Development Fund (CB06/06/1088). UP, VB and MG were funded by Slovenian Research Agency (research core funding No. P3-0427). MIA-A was funded by the Egyptian Government Ph.D. The STOPPA study was funded by the Swedish Research Council project grant 2018-02640 and the Swedish Asthma and Allergy Research Foundation.

Human samples and related data used in this study were provided by the Basque Biobank/Biodonostia Node www.biobancovasco.org and were processed following standard operating procedures with appropriate approval of the relevant Ethics Committee and Scientific Advisory Board.
