# Supplementary material for: Epigenome-Wide Association Studies of the Fractional Exhaled Nitric Oxide and Bronchodilator Drug Response in Moderate-to-Severe Pediatric Asthma
Source: Biomedicines. 2023 Feb 23;11(3):676. doi: 10.3390/biomedicines11030676 (PMC10044864; doi:10.3390/biomedicines11030676)
Supplement: Supplementary file 1 [file biomedicines-11-00676-s001.zip › Supplementary SI_SysPharmPediA_consortium_Author_list_Feb_2023.pdf]

Supplementary SI: SysPharmPediA consortium members (authors list)

Name	Country	Affiliation
Olaia Sardón-Prado, MD, PhD	Spain	Division of Pediatric Respiratory Medicine, Hospital Universitario Donostia, San Sebastián, Spain<br>Department of Pediatrics, University of the Basque Country (UPV/EHU), San Sebastián, Spain.
Paula Corcuera-Elosegui, MD, PhD	Spain	Division of Pediatric Respiratory Medicine, Hospital Universitario Donostia, San Sebastián, Spain.
Javier Korta-Murua, MD, PhD	Spain	Division of Pediatric Respiratory Medicine, Hospital Universitario Donostia, San Sebastián, Spain
Mahmoud I. Abdel-Aziz, PhD	The Netherlands	Department of Respiratory Medicine, Amsterdam UMC, University of Amsterdam, Amsterdam, The Netherlands. Department of Clinical Pharmacy, Faculty of Pharmacy, Assiut University, Assiut, Egypt.
Paul Brinkman, PhD	The Netherlands	Department of Respiratory Medicine, Amsterdam UMC, University of Amsterdam, Amsterdam, The Netherlands.
Susanne J. H. Vijverberg, PhD	The Netherlands	Department of Respiratory Medicine, Amsterdam UMC, University of Amsterdam, Amsterdam, The Netherlands.
Anne H. Neerincx, PhD	The Netherlands	Department of Respiratory Medicine, Amsterdam UMC, University of Amsterdam, Amsterdam, The Netherlands.
Simone Hashimoto, MD, PhD	The Netherlands	Department of Respiratory Medicine, Amsterdam UMC, University of Amsterdam, Amsterdam, The Netherlands. Department of Pediatric Respiratory Medicine, Emma Children's Hospital, Amsterdam UMC, Amsterdam, The Netherlands.
Aletta D. Kraneveld, PhD	The Netherlands	Division of Pharmacology, Utrecht Institute for Pharmaceutical Sciences, Faculty of Science, Utrecht University, Utrecht, the Netherlands.<br>Institute for Risk Assessment Sciences, Faculty of Veterinary Medicine, Utrecht University, Utrecht, the Netherlands.
Anke H. Maitland-van der Zee, PharmD, PhD	The Netherlands	Department of Respiratory Medicine, Amsterdam UMC, University of Amsterdam, Amsterdam, The Netherlands.<br>Department of Pediatric Respiratory Medicine, Emma Children's Hospital, Amsterdam UMC, Amsterdam, The Netherlands.
Maria Pino-Yanes, PhD	Spain	Genomics and Health Group, Department of Biochemistry, Microbiology, Cell Biology and Genetics, Universidad de La Laguna, La Laguna, Santa Cruz de Tenerife, Spain.<br>CIBER de Enfermedades Respiratorias, Instituto de Salud Carlos III, Madrid, Spain.<br>Instituto de Tecnologías Biomédicas (ITB), Universidad de La Laguna, Santa Cruz de Tenerife, Spain.
Esther Herrera-Luis, PhD	Spain	Genomics and Health Group, Department of Biochemistry, Microbiology, Cell Biology and Genetics, Universidad de La Laguna, La Laguna, Santa Cruz de Tenerife, Spain.
Javier Perez-Garcia, PharmD	Spain	Genomics and Health Group, Department of Biochemistry, Microbiology, Cell Biology and Genetics, Universidad de La Laguna, La Laguna, Santa Cruz de Tenerife, Spain.
Mario Martin-Almeida, BSc	Spain	Genomics and Health Group, Department of Biochemistry, Microbiology, Cell Biology and Genetics, Universidad de La Laguna, La Laguna, Santa Cruz de Tenerife, Spain.
Uroš Potočnik, PhD	Slovenia	Center for Human Molecular Genetics and Pharmacogenomics, Faculty of Medicine, University of Maribor, Maribor, Slovenia.

		Laboratory for Biochemistry, Molecular biology and Genomics, Faculty of Chemistry and Chemical Engineering, University of Maribor, Maribor, Slovenia.<br>Department for Science and Research, University Medical Centre Maribor, Maribor, Slovenia
Mario Gorenjak MBI, PhD	Slovenia	Center for Human Molecular Genetics and Pharmacogenomics, Faculty of Medicine, University of Maribor, Maribor, Slovenia.
Vojko Berce MD, PhD	Slovenia	Center for Human Molecular Genetics and Pharmacogenomics, Faculty of Medicine, University of Maribor, Maribor, Slovenia.<br>Clinic of Pediatrics, University Medical Centre Maribor, Maribor, Slovenia.
Maya Petek, PhD	Slovenia	Center for Human Molecular Genetics and Pharmacogenomics, Faculty of Medicine, University of Maribor, Maribor, Slovenia.
Michael Kabesch, MD, PhD	Germany	Department of Pediatric Pneumology and Allergy, University Children’s Hospital Regensburg (KUNO) at the Hospital St. Hedwig of the Order of St. John, University of Regensburg, Regensburg, Germany.<br>Science and Development Campus Regensburg (WECARE), University Children’s Hospital Regensburg (KUNO) at the Hospital St. Hedwig of the Order of St. John, University of Regensburg, Regensburg, Germany.
Susanne Harner	Germany	Department of Pediatric Pneumology and Allergy, University Children’s Hospital Regensburg (KUNO) at the Hospital St. Hedwig of the Order of St. John, University of Regensburg, Regensburg, Germany.
Christine Wolff	Germany	Science and Development Campus Regensburg (WECARE), University Children’s Hospital Regensburg (KUNO) at the Hospital St. Hedwig of the Order of St. John, University of Regensburg, Regensburg, Germany
Antoaneta Toncheva	Germany	Department of Pediatric Pneumology and Allergy, University Children’s Hospital Regensburg (KUNO) at the Hospital St. Hedwig of the Order of St. John, University of Regensburg, Regensburg, Germany.
Susanne Brandstetter	Germany	Science and Development Campus Regensburg (WECARE), University Children’s Hospital Regensburg (KUNO) at the Hospital St. Hedwig of the Order of St. John, University of Regensburg, Regensburg, Germany.
Elisa Valletta	Germany	Science and Development Campus Regensburg (WECARE), University Children’s Hospital Regensburg (KUNO) at the Hospital St. Hedwig of the Order of St. John, University of Regensburg, Regensburg, Germany.
Heike Buntrock-Döpke	Germany	Science and Development Campus Regensburg (WECARE), University Children’s Hospital Regensburg (KUNO) at the Hospital St. Hedwig of the Order of St. John, University of Regensburg, Regensburg, Germany.
Joris C. Verster, PhD	The Netherlands	Division of Pharmacology, Utrecht Institute for Pharmaceutical Sciences, Faculty of Science, Utrecht University, Utrecht, the Netherlands.<br>Institute for Risk Assessment Sciences, Faculty of Veterinary Medicine, Utrecht University, Utrecht, the Netherlands.
Nikki Kerssemakers	The Netherlands	Division of Pharmacology, Utrecht Institute for Pharmaceutical Sciences, Faculty of Science, Utrecht University, Utrecht, the Netherlands.
Catarina Almqvist, MD, PhD	Sweden	Dept of Medical Epidemiology and Biostatistics, Karolinska Institutet, Stockholm, Sweden.<br>Pediatric Allergy and Pulmonology Unit at Astrid Lindgren Children’s Hospital, Karolinska University Hospital.

Anna Hedman, PhD	Sweden	Dept of Medical Epidemiology and Biostatistics, Karolinska Institutet, Stockholm, Sweden.
Mwenya Mubanga, PhD	Sweden	Dept of Medical Epidemiology and Biostatistics, Karolinska Institutet, Stockholm, Sweden.
Tong Gong, PhD	Sweden	Dept of Medical Epidemiology and Biostatistics, Karolinska Institutet, Stockholm, Sweden.
Anne Örtqvist	Sweden	Dept of Medical Epidemiology and Biostatistics, Karolinska Institutet, Stockholm, Sweden.
